# End‐of‐Life Preferences: How Do They Differ Between Individuals with Poor and Normal Cognition?

**DOI:** 10.1002/alz70858_104553

**Published:** 2025-12-26

**Authors:** Zahra Rahemi, Matthew Lee Smith, Juanita‐Dawne Bacsu, Elham Hariri Kashani, Swann Arp Adams

**Affiliations:** ^1^ Clemson University, Greenville, SC, USA; ^2^ Texas A&M University, College Station, TX, USA; ^3^ University of Saskatchewan, Saskatoon, SK, Canada; ^4^ Kashan University of Medical Sciences, Kashan, Isfahan, Iran (Islamic Republic of); ^5^ University of South Carolina, Columbia, SC, USA

## Abstract

**Background:**

Communicating end‐of‐life (EOL) preferences is essential to ensure that care aligns with individuals’ values and priorities, particularly for those with cognitive impairments who may face unique challenges in expressing their wishes. However, little is known about how EOL preferences differ between individuals with different cognition levels, particularly across diverse populations. This study examines these differences, considering variations in race/ethnicity and socioeconomic status.

**Method:**

Public‐use data from the exit files of the Health and Retirement Survey (HRS,1995‐2016) were analyzed. Exit files include participants who died since the previous HRS wave and had a proxy respondent who completed an interview. Cognition status was determined based on the proxy's evaluation of the deceased individual's memory one month before death, ranked on a Likert scale from poor to excellent. Descriptive statistics were calculated, and chi‐square tests were used to assess significant differences between groups.

**Result:**

The decedents’ impaired cognition group was significantly more likely to be single, less educated, white, and female. They were also significantly more likely to have a living will compared to those with normal cognition (46% vs. 42%, *p* <0.01). Conversely, a higher proportion of individuals with normal cognition had discussed EOL care compared to those with impaired cognition (56% vs. 51%, *p* <0.01). No significant differences by cognition status were found for family refusal of treatment or preferences for withholding treatment at EOL. However, significantly fewer participants with impaired cognition expressed a preference for receiving all care (29% vs. 33%, *p* <0.01), while a significantly higher proportion preferred to be made comfortable compared to those with normal cognition (95% vs. 92%, *p* <0.01). Interestingly, no significant differences were observed between the groups regarding whether cost influenced their EOL decisions.

**Conclusion:**

Individuals with impaired cognition may differ from those with normal cognition in their end‐of‐life preferences, particularly in their likelihood of having a living will, discussing EOL care, and preferring comfort‐focused care over life‐prolonging measures, highlighting the need for targeted interventions to address cognitive and sociodemographic disparities in advance care planning.

